# SUNCT syndrome treated with gamma knife targeting the trigeminal nerve and sphenopalatine ganglion

**DOI:** 10.1007/s10194-012-0457-2

**Published:** 2012-06-09

**Authors:** Thomas Mathew, Dwarakanath Srinivas, Sushanth Aroor, Chandrajit Prasad, Sampath Somanna, Raghunandan Nadig, G. R. K. Sarma

**Affiliations:** 1 St. John’s Medical College, Bangalore, India; 2 NIMHANS, Bangalore, India

SUNCT syndrome, an abbreviation for short lasting-unilateral neuralgiform headache attacks with conjunctival injection and tearing is one of the most debilitating unilateral headache syndromes often refractory to medical therapy [1]. We report a case of a 50-year-old man diagnosed with refractory SUNCT syndrome having a near complete response to gamma knife surgery targeting the trigeminal nerve and sphenopalatine ganglion.

A 50-year-old man came with a 1-year history of severe right-sided episodic stabbing hemifacial pain over the V1 and V2 segments of the right trigeminal nerve. Patient was initially diagnosed as trigeminal neuralgia and was treated with carbamazepine and pregabalin with no response. He was admitted at our institution for detailed evaluation. It was observed that he experienced 100–200 episodes per day, with each episode lasting 10–30 s. Each episode was associated with ipsilateral conjunctival injection and lacrimation. General and neurological examination was unremarkable. A detailed ophthalmic examination and intraocular pressure measurement were normal. Magnetic resonance imaging of the brain and magnetic resonance angiogram of the intracranial vessels were normal. Once a diagnosis of SUNCT syndrome was made, a trial of lamotrigine was given. On the third week of lamotrigine, the patient developed skin rashes and lamotrigine was stopped. He was put on a combination of topiramate and gabapentin for about 1 month without any benefit. The patient was in excruciating pain and had even contemplated suicide. As a last resort, a surgical option for the treatment was considered. The patient was planned for gamma knife radiosurgery after explaining the procedure and possible outcomes. A Leksell Frame was fixed under local anesthesia. A 1-mm thick axial T1 contrast enhanced magnetization prepared rapid gradient echo (MPR) was performed. Both the trigeminal nerve and the sphenopalatine ganglion were targeted. The trigeminal nerve was targeted at the root exit zone with a total dose of 80 Gy to the 100 % isodose using a single 4-mm collimator. The same dose using a single 8-mm collimator was used to target the sphenopalatine ganglion. The procedure was uneventful. Less than 1 % of the pons received 5.6 Gy and 0 % received 6 Gy or higher which were well within the radiation safety limits. No point of the pons received more than 4.8 Gy. 0 % of the internal carotid artery received 7 Gy while the point dose at the part of the ICA closest to the isodose line received a maximum of 4.3 Gy.

The patient was totally free of pain within the first day of surgery. At 4 months of follow-up, he had no spontaneous attacks but had very occasional brief episodes precipitated by touching of the periorbital region.

While the success of stereotactic surgery in the treatment of trigeminal neuralgia is well known [2], its effectiveness in treatment for trigeminal autonomic cephalgias is yet to be established. A recent study showed that gamma knife therapy was successful in 60 % of the cases of intractable medically refractory cluster headache [3]. A report of two cases of medical refractory SUNCT in 2002 had a poor response to gamma knife surgery [4]. However, the surgery targeted only the trigeminal nerve. Only recently a case of SUNCT syndrome successfully treated with gamma knife surgery was reported, in which both the trigeminal nerve and sphenopalatine ganglion were targeted [5]. This is the second case of medically refractory SUNCT syndrome in which gamma knife surgery of both trigeminal nerve and sphenopalatine ganglion yielded a near total relief to the patient. We believe that the inclusion of the sphenopalatine ganglion appears to be important for the favorable outcome of surgery, in view of the autonomic involvement in SUNCT syndrome.
